# Supplementary material for: Maximization of Open Hospital Capacity under Shortage of SARS-CoV-2 Vaccines—An Open Access, Stochastic Simulation Tool
Source: Vaccines (Basel). 2021 May 22;9(6):546. doi: 10.3390/vaccines9060546 (PMC8224649; doi:10.3390/vaccines9060546)
Supplement: Supplementary file 1 [file vaccines-09-00546-s001.zip › vaccines-1201874-SI.pdf]

```
Sub vaccination_scenario_modules()



        '++++++++++++++++++++++++++++++++++++++++++++
        'copyrights and license


        'Copyright 2021 Wolfram A Bosbach, PhD

            'This program is free software: you can redistribute it and/or modify
            'it under the terms of the GNU General Public License as published by
            'the Free Software Foundation, either version 3 of the License, or
            'any later version.

            'This program is distributed in the hope that it will be useful,
            'but WITHOUT ANY WARRANTY; without even the implied warranty of
            'MERCHANTABILITY or FITNESS FOR A PARTICULAR PURPOSE.  See the
            'GNU General Public License for more details.

            'You should have received a copy of the GNU General Public License
            'along with this program.  If not, see <https://www.gnu.org/licenses/>.

        '++++++++++++++++++++++++++++++++++++++++++++
        'vba and xlsm files are deposited for open access at:
                'DOI: 10.5281/zenodo.4589333
        '++++++++++++++++++++++++++++++++++++++++++++



Dim Staff_Iterator As String
Dim Infected, Symptoms, Test_Positive, Infectious, Vaccinated, Unit, Unit_Count
Dim New_Infected, New_Symptoms, New_Test_Positive, New_Infectious, New_Vaccinated, New_Unit, New_Unit_Count
```

```
Dim String_Split As String
Dim New_Staff_Status, Staff_Status_yesday
   'array entries
     'Staff_Status(0)  :  days since infected
     'Staff_Status(1)  :  symptomatic
     'Staff_Status(2)  :  positive in tests
     'Staff_Status(3)  :  infectious for environment
     'Staff_Status(4)  :  number vaccinations
     'Staff_Status(5)  :  assigned unit
     'Staff_Status(6)  :  staff count on unit
     'Staff_Status(7)  :  staff age
     'Staff_Status(8)  :
     'Staff_Status(9)  :
     'Staff_Status(10) :
     '...
'parameter variation
    Dim v_11, v_12, v_21, v_22, v_23, v_24, v_25, v_26
    Dim v_11_start, v_12_start, v_21_start, v_22_start, v_23_start, v_24_start, v_25_start, v_26_start
    Dim v_11_stop, v_12_stop, v_21_stop, v_22_stop, v_23_stop, v_24_stop, v_25_stop, v_26_stop
    Dim n, swap



'total counters
    Dim Chief, Executive_Senior_Physician, Senior_Physician, Physician, Nurse As Integer
'counters per unit
    Dim AandE_Phys, Ward_1_Phys, Ward_2_Phys, Ward_3_Phys As Integer
    Dim AandE_Senior, Ward_1_Senior, Ward_2_Senior, Ward_3_Senior As Integer
    Dim AandE_ExecSenior, Ward_1_ExecSenior, Ward_2_ExecSenior, Ward_3_ExecSenior As Integer
    Dim AandE_Nurse, Ward_1_Nurse, Ward_2_Nurse, Ward_3_Nurse As Integer
'positive, non-symptomatic staff per unit
    Dim Staff_pos_nonSymptomatic_AandE, Staff_pos_nonSymptomatic_AandE_yesday As Integer
    Dim Staff_pos_nonSymptomatic_ward_1, Staff_pos_nonSymptomatic_ward_1_yesday As Integer
    Dim Staff_pos_nonSymptomatic_ward_2, Staff_pos_nonSymptomatic_ward_2_yesday As Integer
    Dim Staff_pos_nonSymptomatic_ward_3, Staff_pos_nonSymptomatic_ward_3_yesday As Integer
'infection events
```

```
    Dim Infection_event_AandE, Infection_event_Ward_1, Infection_event_Ward_2, Infection_event_Ward_3 As Integer


'unit operational
    Dim AandE_open, Ward_1_open, Ward_2_open, Ward_3_open
'rollout plan
    Dim Staff_counter, Vaccines_today As Integer
    Dim AE_not_vaccinated, AE_not_vaccinated_yesday As Integer


'vaccination rollout plans
    Dim Rollout_plan(3)
        Rollout_plan(0) = 1  '1 : hierarchy top down
        Rollout_plan(1) = 2  '2 : exposure focused
    'with units per day
    Dim Rollout_rate(10)
        Rollout_rate(0) = 0 'vaccines applied to staff per day
        Rollout_rate(1) = 1
        Rollout_rate(2) = 2
        Rollout_rate(3) = 5
        Rollout_rate(4) = 7
        Rollout_rate(5) = 10
        Rollout_rate(6) = 20
        Rollout_rate(7) = 30
        Rollout_rate(8) = 40


'exposure modelling
    Dim AE_pat_pos(10)
        AE_pat_pos(0) = 0 'positive rate A&E patients
        AE_pat_pos(1) = 0.1
        AE_pat_pos(2) = 0.25
        AE_pat_pos(3) = 0.5
        AE_pat_pos(4) = 0.75
        AE_pat_pos(5) = 0.9
        AE_pat_pos(6) = 1
    Dim AE_pat_shift As Integer
```

```
        AE_pat_shift = 20 'patients per shift on A&E
    Dim Transmission, Probab
    Dim R_S(10)
        R_S(0) = 0.1     'R_S as used by TU Berlin, DOI: 10.14279/depositonce-11387
        R_S(1) = 0.5     'in model, risk shared by 5 people
        R_S(2) = 1
        R_S(3) = 2
        R_S(4) = 4
        R_S(5) = 7
        R_S(6) = 10
        R_S(7) = 14
        R_S(8) = 20
    Dim Pat_Contact_factor(5)
        Pat_Contact_factor(0) = 1 'factor of reduced contact of chief and senior executives to patients and unit
staff
        Pat_Contact_factor(1) = 2
        Pat_Contact_factor(2) = 5
        Pat_Contact_factor(3) = 10
        Pat_Contact_factor(4) = 20


'disease time parameters
    Dim t_incubate(5)
        t_incubate(0) = 0 ' time between transmission and clinical symptoms
        t_incubate(1) = 5
    Dim t_recovery(5)
        t_recovery(0) = 1 'time after which staff returns to work
        t_recovery(1) = 5
        t_recovery(2) = 10
        t_recovery(3) = 14
        t_recovery(4) = 20

'staff age
    Dim Age As Integer
    Dim Age_Factor(10)
        Age_Factor(0) = 1 'define by what factor transmission risk increases for 65 years of age, compared to 18
years
```

```
        Age_Factor(1) = 1.5
        Age_Factor(2) = 2.5
        Age_Factor(3) = 5
        Age_Factor(4) = 10
        Age_Factor(5) = 20

'saving path
    Dim Path_xlsm(10, 10, 10, 5, 5, 10, 5)


        '-----------------------------------------
        'clear statistical data in table 6 and 7
            Tabelle6.Activate
            Tabelle6.Range("D119:CY50000").Select
            Selection.ClearContents
            Tabelle7.Activate
            Tabelle7.Range("D119:CY50000").Select
            Selection.ClearContents
            Tabelle2.Activate

'#####################################################################
'build vaccination model iterator
    'rollout
        'v_11 : rollout plan
        'v_12 : rollout rate per day
    'exposure
        'v_21 : positive rate A&E patients
        'v_22 : R_S as used by TU Berlin
        'v_23 : factor of reduced contact of chief and senior executives to patients and unit staff
        'v_24 : incubation time
        'v_25 : age factor, transmission risk increases for 65 years of age, compared to 18 years
        'v_26 : 'time after which staff returns to work

    'define saving path
            v_12 = 0
            Do 'loop v_12 - rollout rate per day
                v_21 = 0
```

```
            Do 'loop v_21 - positive rate A&E patients
                v_22 = 0
                Do 'loop v_22 - R_S as used by TU Berlin
                    v_23 = 0
                    Do 'loop v_23 - factor of reduced contact of chief and senior executives to patients and
unit staff
                        v_24 = 0
                        Do 'loop v_24 - incubation time
                            v_25 = 0
                            Do 'loop v_25 - age factor, transmission risk increases for 65 years of age,
compared to 18 years
                                v_26 = 0
                                Do 'loop v_26 - recovery time
                                    'create path for saving model run
                                    Path_xlsm(v_12, v_21, v_22, v_23, v_24, v_25, v_26) = _
                                        "daily_rate_" & Rollout_rate(v_12) _
                                        & "_AE_pospat_" & AE_pat_pos(v_21) & "_R_s_" & R_S(v_22) _
                                        & "_contact_fac_" & Pat_Contact_factor(v_23) & "_incubate_" &
t_incubate(v_24) _

                                        & "_age_fac_" & Age_Factor(v_25) & "_t_recovery_" & t_recovery(v_26)
                                    Path_xlsm(v_12, v_21, v_22, v_23, v_24, v_25, v_26) = _
                                        "C:\400 Heidelberg\200 projects\02_hos_op_mgm\03 parameter studies\" _
                                        & Path_xlsm(v_12, v_21, v_22, v_23, v_24, v_25, v_26)
                                v_26 = v_26 + 1
                                Loop While v_26 <= 4
                            v_25 = v_25 + 1
                            Loop While v_25 <= 5
                        v_24 = v_24 + 1
                        Loop While v_24 <= 1
                    v_23 = v_23 + 1
                    Loop While v_23 <= 4
                v_22 = v_22 + 1
                Loop While v_22 <= 8
            v_21 = v_21 + 1
            Loop While v_21 <= 6
        v_12 = v_12 + 1
        Loop While v_12 <= 8
```

```
'boundaries for parameter variation
v_11_start = 0
v_12_start = 1
v_21_start = 2
v_22_start = 5
v_23_start = 2
v_24_start = 1
v_25_start = 2
v_26_start = 2
v_11_stop = 1
v_12_stop = 1
v_21_stop = 2
v_22_stop = 5
v_23_stop = 2
v_24_stop = 1
v_25_stop = 2
v_26_stop = 2

    'parameters base case
    'v_11_start = 0
    'v_12_start = 1
    'v_21_start = 2
    'v_22_start = 5
    'v_23_start = 2
    'v_24_start = 1
    'v_25_start = 2
    'v_26_start = 2
    'v_11_stop = 1
    'v_12_stop = 1
    'v_21_stop = 2
    'v_22_stop = 5
    'v_23_stop = 2
    'v_24_stop = 1
    'v_25_stop = 2
    'v_26_stop = 2
```

```
'loop vaccination model for parameter variation
    v_12 = v_12_start
    Do 'loop v_12 - rollout rate per day
        v_21 = v_21_start
        Do 'loop v_21 - positive rate A&E patients
            v_22 = v_22_start
            Do 'loop v_22 - R_S as used by TU Berlin
                v_23 = v_23_start
                Do 'loop v_23 - factor of reduced contact of chief and senior executives to patients and unit staff
                    v_24 = v_24_start
                    Do 'loop v_24 - incubation time
                        v_25 = v_25_start
                        Do 'loop v_25 - age factor, transmission risk increases for 65 years of age, compared to 18
years
                            v_26 = v_26_start
                            Do 'loop v_26 - recovery time
                                'optimise excel
                                Application.ScreenUpdating = False
                                'in each run, do both rollout plans
                                v_11 = v_11_start
                                Do 'loop v_11 - rollout plan




                    '########################################################################
                    'loop around hospital model, too even out statistical outliers
                    n = 1
                    Do While n <= 500
```

```
'==========================================================================================
                                'module-1: define staff age once at t=1

                                        i = 43
                                        Staff_Iterator = Tabelle2.Cells(i, 1).Value
                                        Do While Staff_Iterator <> ""
                                            '-------------------------------------------
                                            'set age by hierarchy group
                                                'nurses
                                                If Staff_Iterator = "Nurse" Then
                                                    Age = WorksheetFunction.RandBetween(18, 65)
                                                'chief
                                                ElseIf Staff_Iterator = "Chief" Then
                                                    Age = WorksheetFunction.RandBetween(51, 65)
                                                'senior executives
                                                ElseIf Staff_Iterator = "Executive_Senior_Physician" Then
                                                    Age = WorksheetFunction.RandBetween(41, 50)
                                                'seniors
                                                ElseIf Staff_Iterator = "Senior_Physician" Then
                                                    Age = WorksheetFunction.RandBetween(33, 40)
                                                'physician
                                                ElseIf Staff_Iterator = "Physician" Then
                                                    Age = WorksheetFunction.RandBetween(25, 32)
                                                End If

'------------------------------------------------------------------------------------------------------------------------
-------
                                            'get staff status @ t=0
                                            t = 0
                                            String_Split = Tabelle2.Cells(i, t + 3).Value
                                            Staff_Status_yesday = Split(String_Split, ",")

                                            'read out staff status @ t-1
                                            Infected = Staff_Status_yesday(0)
                                            Symptoms = Staff_Status_yesday(1)
                                            Test_Positive = Staff_Status_yesday(2)
                                            Infectious = Staff_Status_yesday(3)
```

```
                                            Vaccinated = Staff_Status_yesday(4)
                                            Unit = Staff_Status_yesday(5)
                                            Unit_Count = Staff_Status_yesday(6)

'-----------------------------------------------------------------------------------------------------------
-------
                                                '.....................
                                                    'build new staff status @ t=1
                                                        t = 1
                                                    New_Staff_Status = Infected & "," & Symptoms & "," &
Test_Positive & "," & Infectious & "," & Vaccinated & "," & Unit & "," & Unit_Count & "," & Age
                                                    Tabelle2.Cells(i, t + 3).Value = New_Staff_Status
                                                '.....................

                                        i = i + 1 'loop to next staff member
                                        Staff_Iterator = Tabelle2.Cells(i, 1).Value
                                        Loop


'=================================================================================


                                'calculate staff development over time
                                t = 1
                                Do While t <= 100

                                    'add timeline
                                    Tabelle2.Cells(26, t + 3).Value = t
                                    Tabelle2.Cells(42, t + 3).Value = t
                                    Tabelle6.Cells(5, t + 3).Value = t
                                    Tabelle6.Cells(53, t + 3).Value = t
                                    'reset total staff counter
                                    Chief = 0
                                    Executive_Senior_Physician = 0
                                    Senior_Physician = 0
```

```
                                    Physician = 0
                                    Nurse = 0
                                    'reset counters per unit
                                    AandE_Nurse = 0
                                    Ward_1_Nurse = 0
                                    Ward_2_Nurse = 0
                                    Ward_3_Nurse = 0
                                    AandE_Phys = 0
                                    Ward_1_Phys = 0
                                    Ward_2_Phys = 0
                                    Ward_3_Phys = 0
                                    AandE_ExecSenior = 0
                                    Ward_1_ExecSenior = 0
                                    Ward_2_ExecSenior = 0
                                    Ward_3_ExecSenior = 0
                                    AandE_Senior = 0
                                    Ward_1_Senior = 0
                                    Ward_2_Senior = 0
                                    Ward_3_Senior = 0
                                    'reset vaccines of the day
                                    Vaccines_today = 0


                                    'loop for each time step (dt = 1d) entire staff matrix 3 times



'===========================================================================================
                                        'module-2 : vaccination rollout

                                            'get staff yesterday not vaccinated on A&E
                                            AE_not_vaccinated_yesday = AE_not_vaccinated
                                            AE_not_vaccinated = 0
                                                'new value for AE_not_vaccinated set in module-4 after unit
assignment
                                            'at t=0 all A&E staff not vaccinated
                                            If t = 1 Then
```

```
                                                  AE_not_vaccinated_yesday = 
Application.WorksheetFunction.CountIf(Range("C43:C2000"), "*" & "A&E" & "*")
                                              End If

                                          Staff_counter = 1
                                          i = 43
                                          Staff_Iterator = Tabelle2.Cells(i, 1).Value
                                          Do While Staff_Iterator <> ""


'---------------------------------------------------------------------------------------------------------------------
-------
                                              'get staff status @ t-1
                                              String_Split = Tabelle2.Cells(i, t + 3 - 1).Value
                                              Staff_Status_yesday = Split(String_Split, ",")
                                              'get staff status @ t
                                              String_Split = Tabelle2.Cells(i, t + 3).Value
                                              Staff_Status = Split(String_Split, ",")

                                              'read out staff status @ t-1
                                              Infected = Staff_Status_yesday(0)
                                              Symptoms = Staff_Status_yesday(1)
                                              Test_Positive = Staff_Status_yesday(2)
                                              Infectious = Staff_Status_yesday(3)
                                              Vaccinated = Staff_Status_yesday(4)
                                              Unit = Staff_Status_yesday(5)
                                              Unit_Count = Staff_Status_yesday(6)
                                                  'read out age
                                                  If t = 1 Then 'age only defined from t=1 onwards
                                                      Age = Staff_Status(7)
                                                  Else
                                                      Age = Staff_Status_yesday(7)
                                                  End If
                                              'assume continuation of disease status, amended if vaccine applied to 
staff member
                                              New_Infected = Infected
                                              New_Symptoms = Symptoms
```

```
                                            New_Test_Positive = Test_Positive
                                            New_Infectious = Infectious
                                            'reset new_vaccinated from previous loop
                                            New_Vaccinated = 0


'---------------------------------------------------------------------------------------------------
-------
                                            'if staff member not yet vaccinated and currently non-symptomatic
                                            If Vaccinated = 0 And Symptoms = 0 Then

                                                 '......................
                                                   'rollout plan: top down
                                                   If Rollout_plan(v_11) = 1 Then
                                                       'multiply time by doses per time, check if it is staff
member's turn

                                                       If Staff_counter <= t * Rollout_rate(v_12) Then
                                                           'set member as vaccinated
                                                           New_Vaccinated = 1
                                                           'if during incubation time, assume healing of infection
                                                           New_Infected = 0
                                                           New_Infectious = 0
                                                       End If
                                                   End If
                                                   '......................
                                                   'rollout plan: by exposure
                                                   If Rollout_plan(v_11) = 2 Then
                                                       'add vaccines if still under daily rate
                                                       If Vaccines_today < Rollout_rate(v_12) Then

                                                           'vaccines given to staff by exposure:
                                                               'priority 1 = A&E
                                                               'priority 2 = all other units

                                                           'A&E, by 1st priority
                                                           If Unit = "A&E" Then
```

```
                    New_Vaccinated = 1
                    Vaccines_today = Vaccines_today + 1
                    'if during incubation time, assume healing of
infection
                    New_Infected = 0
                    New_Infectious = 0

                        'debug who gets vaccinated
                        'Tabelle2.Cells(105, t + 3).Value =
Staff_Iterator & "_" & Unit

                End If
                'remaining units with 2nd priority
                If Unit <> "A&E" And AE_not_vaccinated_yesday <
(Rollout_rate(v_12) - Vaccines_today) Then
                    New_Vaccinated = 1
                    Vaccines_today = Vaccines_today + 1
                    'if during incubation time, assume healing of
infection
                    New_Infected = 0
                    New_Infectious = 0

                        'debug who gets vaccinated
                        'Tabelle2.Cells(106, t + 3).Value =
Staff_Iterator & "_" & Unit

                End If

            End If

        End If
        '.....................
        'additional plans possible
            'eg rollout plan: bottom up
```

```
                                                                '.....................
                                                                    'build new staff status
                                                                    New_Staff_Status = New_Infected & "," & New_Symptoms & "," &
New_Test_Positive & "," & New_Infectious & "," & New_Vaccinated & "," & Unit & "," & Unit_Count & "," & Age
                                                                    Tabelle2.Cells(i, t + 3).Value = New_Staff_Status
                                                                '.....................

                                                        Else

'------------------------------------------------------------------------------------------------------------------------
-------
                                                        'copy staff status, if staff member already vaccinated or currenlty
symptomaic
                                                            New_Staff_Status = Tabelle2.Cells(i, t + 3 - 1).Value
                                                            Tabelle2.Cells(i, t + 3).Value = New_Staff_Status
                                                        End If

'------------------------------------------------------------------------------------------------------------------------
-------


                                                    i = i + 1 'loop to next staff member
                                                    Staff_Iterator = Tabelle2.Cells(i, 1).Value
                                                    'and increase staff_counter
                                                    Staff_counter = Staff_counter + 1
                                                    Loop





'===========================================================================================
                                                    'module-3 : disease status

                                                            'shift notvacc to yesterday's variable, and reset variable
                                                            Staff_pos_nonSymptomatic_AandE_yesday =
Staff_pos_nonSymptomatic_AandE
```

```
            Staff_pos_nonSymptomatic_ward_1_yesday =
Staff_pos_nonSymptomatic_ward_1
            Staff_pos_nonSymptomatic_ward_2_yesday =
Staff_pos_nonSymptomatic_ward_2
            Staff_pos_nonSymptomatic_ward_3_yesday =
Staff_pos_nonSymptomatic_ward_3
                'new values for non-symptomatic staff per unit set in loop-3,
after unit assignment

            'reset variables of infection_events
            Infection_event_Ward_1 = 0
            Infection_event_Ward_2 = 0
            Infection_event_Ward_3 = 0
            Infection_event_AandE = 0


        i = 43
        Staff_Iterator = Tabelle2.Cells(i, 1).Value
        Do While Staff_Iterator <> ""


'-----------------------------------------------------------------------------------------------------------------------
-------
            'get staff status @ t-1
            String_Split = Tabelle2.Cells(i, t + 3 - 1).Value
            Staff_Status_yesday = Split(String_Split, ",")
            'get staff status @ t
            String_Split = Tabelle2.Cells(i, t + 3).Value
            Staff_Status = Split(String_Split, ",")

            'read out staff status @ t-1
            Infected = Staff_Status_yesday(0)
            Symptoms = Staff_Status_yesday(1)
            Test_Positive = Staff_Status_yesday(2)
            Infectious = Staff_Status_yesday(3)
            Vaccinated = Staff_Status_yesday(4)
            Unit = Staff_Status_yesday(5)
```

```
Unit_Count = Staff_Status_yesday(6)
'read out staff status @ t
New_Infected = Staff_Status(0)
New_Symptoms = Staff_Status(1)
New_Test_Positive = Staff_Status(2)
New_Infectious = Staff_Status(3)
New_Vaccinated = Staff_Status(4)
New_Unit = Staff_Status(5)
New_Unit_Count = Staff_Status(6)
Age = Staff_Status(7)



'----------------------------------------------------------------------------------------------------------------------
-------
'amend and print staff status @ t, unless vaccinated
If New_Vaccinated = 0 Then

    '......................
    'define New_Infection for staff member
        '1. staff currently infected?
        'add 1 more illness day
        If Infected > 0 Then
            New_Infected = Infected + 1
        End If
        'symptomatic when incubation_time over
        If New_Infected = t_incubate(v_24) Then
            New_Symptoms = 1
        End If
        'assume recovery after t_recovery
                'convert to int
                Infected = Infected * 1
        If Infected = t_incubate(v_24) + t_recovery(v_26) Then
            New_Infected = 0
            New_Infectious = 0
            New_Symptoms = 0
```

```
End If

'2. staff yesterday not infected and coming to work
If Infected = 0 Then
    'staff on A&E
        'exposed to patients, positive at rate of AE_pat_pos
        'exposed to other staff
    If Unit = "A&E" Then
        'new infectious status by exposure during work
        Transmission = WorksheetFunction.RandBetween(0, 100)
        'calculate Probab for A&E situation
            'positive rate patients * patients per shift
            Probab = AE_pat_pos(v_21) * AE_pat_shift
            'add positive, non-symptomatic staff, depending on unit
            Probab = Probab + Staff_pos_nonSymptomatic_AandE_yesday
            'Probab age adjusted:
            '18 -> transmission unchanged
            '65 -> transmission * age_factor
            Probab = Probab _
                   * ((Age_Factor(v_25) - 1) / (65 - 18) * (Age - 18) + 1)
            'Probab adjusted to R_S
            Probab = Probab * R_S(v_22) / 5
                'reduce probability for chief and senExec due to less patient contact
                If Staff_Iterator = "Chief" Or Staff_Iterator = "Executive_Senior_Physician" Then
                     Probab = Probab / Pat_Contact_factor(v_23)
                End If

        'transmission within probab?
        If Transmission <= Probab Then
            'set staff member as  infected
            New_Infected = 1
            New_Infectious = 1
```

```
        End If

    End If

    If Unit <> "A&E" Then
        'new infectious status by exposure during work
        Transmission = WorksheetFunction.RandBetween(0, 100)
        'calculate Probab for A&E situation
            'basic probability at home, false_negative tests
            Probab = 1 / 100
            'positive, non-symptomatic staff, depending on unit
                If Unit = "ward1" Then
                    Probab = Probab + Staff_pos_nonSymptomatic_ward_1_yesday

                ElseIf Unit = "ward2" Then
                    Probab = Probab + Staff_pos_nonSymptomatic_ward_2_yesday

                ElseIf Unit = "ward3" Then
                    Probab = Probab + Staff_pos_nonSymptomatic_ward_3_yesday

                End If
            'Probab age adjusted:
            '18 -> transmission unchanged
            '65 -> transmission * age_factor
            Probab = Probab _
                   * ((Age_Factor(v_25) - 1) / (65 - 18) * (Age - 18) + 1)

            'Probab adjusted to R_S
            Probab = Probab * R_S(v_22) / 5

        'transmission within probab?
        If Transmission <= Probab Then
            'set staff member as  infected
            New_Infected = 1
            New_Infectious = 1
        End If
```

```
        End If

        'infection events
        If New_Infected = 1 Then
            'count infection events by unit
            If Unit = "A&E" Then
                Infection_event_AandE = Infection_event_AandE + 1
            ElseIf Unit = "ward1" Then
                Infection_event_Ward_1 = Infection_event_Ward_1 + 1
            ElseIf Unit = "ward2" Then
                Infection_event_Ward_2 = Infection_event_Ward_2 + 1
            ElseIf Unit = "ward3" Then
                Infection_event_Ward_3 = Infection_event_Ward_3 + 1
            End If
        End If


    End If

        'count infected staff
        If New_Infected > 0 Then
            'count staff infected
            If Staff_Iterator = "Chief" Then
                Chief = Chief + 1
            ElseIf Staff_Iterator = "Executive_Senior_Physician"
Then
                Executive_Senior_Physician =
Executive_Senior_Physician + 1
            ElseIf Staff_Iterator = "Senior_Physician" Then
                Senior_Physician = Senior_Physician + 1
            ElseIf Staff_Iterator = "Physician" Then
                Physician = Physician + 1
            ElseIf Staff_Iterator = "Nurse" Then
                Nurse = Nurse + 1
            End If
        End If
```

```
                                                        '.....................
                                                            'build new staff status
                                                            New_Staff_Status = New_Infected & "," & New_Symptoms & "," &
New_Test_Positive & "," & New_Infectious & "," & New_Vaccinated & "," & Unit & "," & Unit_Count & "," & Age
                                                            Tabelle2.Cells(i, t + 3).Value = New_Staff_Status
                                                        '.....................


                                                    End If

'------------------------------------------------------------------------------------------------------------------
-------


                                        i = i + 1 'loop to next staff member
                                        Staff_Iterator = Tabelle2.Cells(i, 1).Value
                                        Loop





'==========================================================================================
                                        'module-4 : assign staff to units

                                                    'reset counter of infectious, non-symptomatic staff
                                                    Staff_pos_nonSymptomatic_AandE = 0
                                                    Staff_pos_nonSymptomatic_ward_1 = 0
                                                    Staff_pos_nonSymptomatic_ward_2 = 0
                                                    Staff_pos_nonSymptomatic_ward_3 = 0

                                        i = 43
                                        Staff_Iterator = Tabelle2.Cells(i, 1).Value
                                        Do While Staff_Iterator <> ""


'------------------------------------------------------------------------------------------------------------------
```

```
-------
                                            'get staff status @ t-1
                                            String_Split = Tabelle2.Cells(i, t + 3 - 1).Value
                                            Staff_Status_yesday = Split(String_Split, ",")
                                            'get staff status @ t
                                            String_Split = Tabelle2.Cells(i, t + 3).Value
                                            Staff_Status = Split(String_Split, ",")

                                            'read out staff status @ t-1
                                            Infected = Staff_Status_yesday(0)
                                            Symptoms = Staff_Status_yesday(1)
                                            Test_Positive = Staff_Status_yesday(2)
                                            Infectious = Staff_Status_yesday(3)
                                            Vaccinated = Staff_Status_yesday(4)
                                            Unit = Staff_Status_yesday(5)
                                            Unit_Count = Staff_Status_yesday(6)
                                            'read out staff status @ t
                                            New_Infected = Staff_Status(0)
                                            New_Symptoms = Staff_Status(1)
                                            New_Test_Positive = Staff_Status(2)
                                            New_Infectious = Staff_Status(3)
                                            New_Vaccinated = Staff_Status(4)
                                            New_Unit = Staff_Status(5)
                                            New_Unit_Count = Staff_Status(6)
                                            Age = Staff_Status(7)


'-------------------------------------------------------------------------------------------------------------------
-------

                                            'amend and print staff status @ t, unless infected
                                            If New_Symptoms = 0 Then

                                                '.....................
                                                'define New_unit by priority:

                                                        'reset unit variables
```

```
                                        New_Unit = 0
                                        New_Unit_Count = 0

                                    '3.1 chief
                                    If Staff_Iterator = "Chief" Then
                                            'chief sent to A&E
                                            AandE_ExecSenior = 1
                                                'set new unit and counter
                                                New_Unit = "A&E"
                                                New_Unit_Count = Unit_Count
                                    End If
                                    '3.2 senior execs
                                    If Staff_Iterator = "Executive_Senior_Physician" Then
                                            'update staff per unit by priority
                                                        'ward_3
                                                        If AandE_ExecSenior = 1 And
Ward_1_ExecSenior = 1 And Ward_2_ExecSenior = 1 And Ward_3_ExecSenior = 0 Then
                                                            Ward_3_ExecSenior = Ward_3_ExecSenior +
1

                                                            'set new_unit and new_unit_count
                                                            New_Unit = "ward3"
                                                            New_Unit_Count = Ward_3_ExecSenior
                                                        End If
                                                    'ward_2
                                                    If AandE_ExecSenior = 1 And Ward_1_ExecSenior =
1 And Ward_2_ExecSenior = 0 And Ward_3_ExecSenior = 0 Then
                                                        Ward_2_ExecSenior = Ward_2_ExecSenior + 1
                                                            'set new_unit and new_unit_count
                                                            New_Unit = "ward2"
                                                            New_Unit_Count = Ward_2_ExecSenior
                                                    End If
                                                'ward_1
                                                If AandE_ExecSenior = 1 And Ward_1_ExecSenior = 0
And Ward_2_ExecSenior = 0 And Ward_3_ExecSenior = 0 Then
                                                    Ward_1_ExecSenior = Ward_1_ExecSenior + 1
                                                            'set new_unit and new_unit_count
                                                            New_Unit = "ward1"
```

```
                                New_Unit_Count = Ward_1_ExecSenior
                        End If
                    'A&E
                    If AandE_ExecSenior = 0 And Ward_1_ExecSenior = 0 And
Ward_2_ExecSenior = 0 And Ward_3_ExecSenior = 0 Then
                        AandE_ExecSenior = AandE_ExecSenior + 1
                                'set new_unit and new_unit_count
                                New_Unit = "A&E"
                                New_Unit_Count = AandE_ExecSenior
                    End If

                End If
                '3.3 seniors
                If Staff_Iterator = "Senior_Physician" Then
                    'update staff per unit by priority
                            'ward_3
                            If AandE_Senior = 1 And Ward_1_Senior = 1
And Ward_2_Senior = 1 And Ward_3_Senior = 0 Then
                                Ward_3_Senior = Ward_3_Senior + 1
                                'set new_unit and new_unit_count
                                New_Unit = "ward3"
                                New_Unit_Count = Ward_3_Senior
                            End If
                        'ward_2
                        If AandE_Senior = 1 And Ward_1_Senior = 1 And
Ward_2_Senior = 0 And Ward_3_Senior = 0 Then
                            Ward_2_Senior = Ward_2_Senior + 1
                                'set new_unit and new_unit_count
                                New_Unit = "ward2"
                                New_Unit_Count = Ward_2_Senior
                        End If
                    'ward_1
                    If AandE_Senior = 1 And Ward_1_Senior = 0 And
Ward_2_Senior = 0 And Ward_3_Senior = 0 Then
                        Ward_1_Senior = Ward_1_Senior + 1
                                'set new_unit and new_unit_count
                                New_Unit = "ward1"
```

```
                        New_Unit_Count = Ward_1_Senior
                End If
            'A&E
            If AandE_Senior = 0 And Ward_1_Senior = 0 And
Ward_2_Senior = 0 And Ward_3_Senior = 0 Then
                AandE_Senior = AandE_Senior + 1
                        'set new_unit and new_unit_count
                        New_Unit = "A&E"
                        New_Unit_Count = AandE_Senior
            End If

        End If
        '3.4 physicians
        If Staff_Iterator = "Physician" Then
            'update staff per unit by priority
                    'ward_3
                    If AandE_Phys = 4 And Ward_1_Phys = 2 And
Ward_2_Phys = 2 And Ward_3_Phys <= 1 Then
                        Ward_3_Phys = Ward_3_Phys + 1
                        'set new_unit and new_unit_count
                        New_Unit = "ward3"
                        New_Unit_Count = Ward_3_Phys
                    End If
                'ward_2
                If AandE_Phys = 4 And Ward_1_Phys = 2 And
Ward_2_Phys <= 1 And Ward_3_Phys = 0 Then
                    Ward_2_Phys = Ward_2_Phys + 1
                        'set new_unit and new_unit_count
                        New_Unit = "ward2"
                        New_Unit_Count = Ward_2_Phys
                End If
            'ward_1
            If AandE_Phys = 4 And Ward_1_Phys <= 1 And
Ward_2_Phys = 0 And Ward_3_Phys = 0 Then
                Ward_1_Phys = Ward_1_Phys + 1
                        'set new_unit and new_unit_count
                        New_Unit = "ward1"
```

```
                                                                        New_Unit_Count = Ward_1_Phys
                                                            End If
                                                        'A&E
                                                        If AandE_Phys <= 4 And Ward_1_Phys = 0 And Ward_2_Phys =
0 And Ward_3_Phys = 0 Then
                                                            AandE_Phys = AandE_Phys + 1
                                                                        'set new_unit and new_unit_count
                                                                        New_Unit = "A&E"
                                                                        New_Unit_Count = AandE_Phys
                                                        End If

                                                End If
                                                '3.5 nurses
                                                If Staff_Iterator = "Nurse" Then
                                                        'update staff per unit by priority
                                                                    'ward_3
                                                                    If AandE_Nurse = 10 And Ward_1_Nurse = 5 And
Ward_2_Nurse = 5 And Ward_3_Nurse < 5 Then
                                                                        Ward_3_Nurse = Ward_3_Nurse + 1
                                                                        'set new_unit and new_unit_count
                                                                        New_Unit = "ward3"
                                                                        New_Unit_Count = Ward_3_Nurse
                                                                    End If
                                                                'ward_2
                                                                If AandE_Nurse = 10 And Ward_1_Nurse = 5 And
Ward_2_Nurse < 5 And Ward_3_Nurse = 0 Then
                                                                    Ward_2_Nurse = Ward_2_Nurse + 1
                                                                        'set new_unit and new_unit_count
                                                                        New_Unit = "ward2"
                                                                        New_Unit_Count = Ward_2_Nurse
                                                                End If
                                                            'ward_1
                                                            If AandE_Nurse = 10 And Ward_1_Nurse < 5 And
Ward_2_Nurse = 0 And Ward_3_Nurse = 0 Then
                                                                Ward_1_Nurse = Ward_1_Nurse + 1
                                                                        'set new_unit and new_unit_count
                                                                        New_Unit = "ward1"
```

```
                                                            New_Unit_Count = Ward_1_Nurse
                                                End If
                                            'A&E
                                            If AandE_Nurse < 10 And Ward_1_Nurse = 0 And
Ward_2_Nurse = 0 And Ward_3_Nurse = 0 Then
                                                AandE_Nurse = AandE_Nurse + 1
                                                            'set new_unit and new_unit_count
                                                            New_Unit = "A&E"
                                                            New_Unit_Count = AandE_Nurse
                                            End If

                                    End If


                            Else 'ie staff member is symptomatic
                                'dont assign staff to unit
                                New_Unit = 0
                                New_Unit_Count = 0
                            End If
                                '.....................
                                'check each day if staff on A&E and not vaccinated
                                If Unit = "A&E" And New_Vaccinated = 0 Then
                                    'invrease counter for AE_not_vaccinated
                                    AE_not_vaccinated = AE_not_vaccinated + 1
                                End If
                                '.....................
                                    'build new staff status
                                    New_Staff_Status = New_Infected & "," & New_Symptoms & "," &
New_Test_Positive & "," & New_Infectious & "," & New_Vaccinated & "," & New_Unit & "," & New_Unit_Count & "," & Age
                                    Tabelle2.Cells(i, t + 3).Value = New_Staff_Status
                                '.....................
                                    'update number: new_infectious, non-symptomatic staff, for each
new_unit

                                    If New_Infected > 0 And New_Symptoms = 0 Then
                                        If New_Unit = "A&E" Then
                                            Staff_pos_nonSymptomatic_AandE = _
                                                Staff_pos_nonSymptomatic_AandE + 1
```

```
                                                                ElseIf New_Unit = "ward1" Then
                                                                    Staff_pos_nonSymptomatic_ward_1 = _
                                                                        Staff_pos_nonSymptomatic_ward_1 + 1
                                                                ElseIf New_Unit = "ward2" Then
                                                                    Staff_pos_nonSymptomatic_ward_2 = _
                                                                        Staff_pos_nonSymptomatic_ward_2 + 1
                                                                ElseIf New_Unit = "ward3" Then
                                                                    Staff_pos_nonSymptomatic_ward_3 = _
                                                                        Staff_pos_nonSymptomatic_ward_3 + 1
                                                                End If
                                                            End If
                                                        '......................

'-----------------------------------------------------------------------------------------------------------------------
-------



                                        i = i + 1 'loop to next staff member
                                        Staff_Iterator = Tabelle2.Cells(i, 1).Value
                                        Loop

'===========================================================================================





                                    '###############################
                                    '5. for each time step:
                                    '5.1 update total staff count in timeline
                                        '5.1.1 for top down rollout
                                        If v_11 = 0 Then
                                                swap = Tabelle2.Cells(27, t + 3).Value
                                                    If n = 1 Then
                                                        swap = 0
                                                    End If
```

```
        Tabelle2.Cells(27, t + 3).Value = swap + Chief
            swap = Tabelle2.Cells(27 + 1, t + 3).Value
                If n = 1 Then
                    swap = 0
                End If
        Tabelle2.Cells(28, t + 3).Value = swap + Executive_Senior_Physician
            swap = Tabelle2.Cells(27 + 2, t + 3).Value
                If n = 1 Then
                    swap = 0
                End If
        Tabelle2.Cells(29, t + 3).Value = swap + Senior_Physician
            swap = Tabelle2.Cells(27 + 3, t + 3).Value
                If n = 1 Then
                    swap = 0
                End If
        Tabelle2.Cells(30, t + 3).Value = swap + Physician
            swap = Tabelle2.Cells(27 + 4, t + 3).Value
                If n = 1 Then
                    swap = 0
                End If
        Tabelle2.Cells(31, t + 3).Value = swap + Nurse
    '5.1.2 for exposure focused rollout
    ElseIf v_11 = 1 Then
            swap = Tabelle2.Cells(12, t + 3).Value
                If n = 1 Then
                    swap = 0
                End If
        Tabelle2.Cells(12, t + 3).Value = swap + Chief
            swap = Tabelle2.Cells(12 + 1, t + 3).Value
                If n = 1 Then
                    swap = 0
                End If
        Tabelle2.Cells(12 + 1, t + 3).Value = swap + Executive_Senior_Physician
            swap = Tabelle2.Cells(12 + 2, t + 3).Value
                If n = 1 Then
                    swap = 0
                End If
```

```
            Tabelle2.Cells(12 + 2, t + 3).Value = swap + Senior_Physician
                swap = Tabelle2.Cells(12 + 3, t + 3).Value
                    If n = 1 Then
                        swap = 0
                    End If
            Tabelle2.Cells(12 + 3, t + 3).Value = swap + Physician
                swap = Tabelle2.Cells(12 + 4, t + 3).Value
                    If n = 1 Then
                        swap = 0
                    End If
            Tabelle2.Cells(12 + 4, t + 3).Value = swap + Nurse
        End If

    '....................................
    '5.2 update unit staff count in unit timeline
        '5.2.1 v_11=0, top down rollout
        If v_11 = 0 Then
                'nurses
                swap = Tabelle6.Cells(9, t + 3).Value
                    If n = 1 Then
                        swap = 0
                    End If
                Tabelle6.Cells(9, t + 3).Value = AandE_Nurse + swap
                swap = Tabelle6.Cells(13, t + 3).Value
                    If n = 1 Then
                        swap = 0
                    End If
                Tabelle6.Cells(13, t + 3).Value = Ward_1_Nurse + swap
                swap = Tabelle6.Cells(17, t + 3).Value
                    If n = 1 Then
                        swap = 0
                    End If
                Tabelle6.Cells(17, t + 3).Value = Ward_2_Nurse + swap
                swap = Tabelle6.Cells(21, t + 3).Value
                    If n = 1 Then
                        swap = 0
                    End If
```

```
    Tabelle6.Cells(21, t + 3).Value = Ward_3_Nurse + swap
'physicians
swap = Tabelle6.Cells(9 - 1, t + 3).Value
    If n = 1 Then
        swap = 0
    End If
Tabelle6.Cells(9 - 1, t + 3).Value = AandE_Phys + swap
swap = Tabelle6.Cells(13 - 1, t + 3).Value
    If n = 1 Then
        swap = 0
    End If
Tabelle6.Cells(13 - 1, t + 3).Value = Ward_1_Phys + swap
swap = Tabelle6.Cells(17 - 1, t + 3).Value
    If n = 1 Then
        swap = 0
    End If
Tabelle6.Cells(17 - 1, t + 3).Value = Ward_2_Phys + swap
swap = Tabelle6.Cells(21 - 1, t + 3).Value
    If n = 1 Then
        swap = 0
    End If
Tabelle6.Cells(21 - 1, t + 3).Value = Ward_3_Phys + swap
    'seniors
    swap = Tabelle6.Cells(9 - 2, t + 3).Value
        If n = 1 Then
            swap = 0
        End If
    Tabelle6.Cells(9 - 2, t + 3).Value = AandE_Senior + swap
    swap = Tabelle6.Cells(13 - 2, t + 3).Value
        If n = 1 Then
            swap = 0
        End If
    Tabelle6.Cells(13 - 2, t + 3).Value = Ward_1_Senior + swap
    swap = Tabelle6.Cells(17 - 2, t + 3).Value
        If n = 1 Then
            swap = 0
        End If
```

```
            Tabelle6.Cells(17 - 2, t + 3).Value = Ward_2_Senior + swap
            swap = Tabelle6.Cells(21 - 2, t + 3).Value
                If n = 1 Then
                    swap = 0
                End If
            Tabelle6.Cells(21 - 2, t + 3).Value = Ward_3_Senior + swap
        'senior executives
        swap = Tabelle6.Cells(9 - 3, t + 3).Value
                If n = 1 Then
                    swap = 0
                End If
        Tabelle6.Cells(9 - 3, t + 3).Value = AandE_ExecSenior + swap
        swap = Tabelle6.Cells(13 - 3, t + 3).Value
                If n = 1 Then
                    swap = 0
                End If
        Tabelle6.Cells(13 - 3, t + 3).Value = Ward_1_ExecSenior + swap
        swap = Tabelle6.Cells(17 - 3, t + 3).Value
                If n = 1 Then
                    swap = 0
                End If
        Tabelle6.Cells(17 - 3, t + 3).Value = Ward_2_ExecSenior + swap
        swap = Tabelle6.Cells(21 - 3, t + 3).Value
                If n = 1 Then
                    swap = 0
                End If
        Tabelle6.Cells(21 - 3, t + 3).Value = Ward_3_ExecSenior + swap
    '5.1.2 v_11=1. exposure focused rolout
    ElseIf v_11 = 1 Then
            'nurses
            swap = Tabelle7.Cells(9, t + 3).Value
                If n = 1 Then
                    swap = 0
                End If
            Tabelle7.Cells(9, t + 3).Value = AandE_Nurse + swap
            swap = Tabelle7.Cells(13, t + 3).Value
                If n = 1 Then
```

```
                swap = 0
            End If
        Tabelle7.Cells(13, t + 3).Value = Ward_1_Nurse + swap
        swap = Tabelle7.Cells(17, t + 3).Value
            If n = 1 Then
                swap = 0
            End If
        Tabelle7.Cells(17, t + 3).Value = Ward_2_Nurse + swap
        swap = Tabelle7.Cells(21, t + 3).Value
            If n = 1 Then
                swap = 0
            End If
        Tabelle7.Cells(21, t + 3).Value = Ward_3_Nurse + swap
    'physicians
    swap = Tabelle7.Cells(9 - 1, t + 3).Value
        If n = 1 Then
            swap = 0
        End If
    Tabelle7.Cells(9 - 1, t + 3).Value = AandE_Phys + swap
    swap = Tabelle7.Cells(13 - 1, t + 3).Value
        If n = 1 Then
            swap = 0
        End If
    Tabelle7.Cells(13 - 1, t + 3).Value = Ward_1_Phys + swap
    swap = Tabelle7.Cells(17 - 1, t + 3).Value
        If n = 1 Then
            swap = 0
        End If
    Tabelle7.Cells(17 - 1, t + 3).Value = Ward_2_Phys + swap
    swap = Tabelle7.Cells(21 - 1, t + 3).Value
        If n = 1 Then
            swap = 0
        End If
    Tabelle7.Cells(21 - 1, t + 3).Value = Ward_3_Phys + swap
        'seniors
        swap = Tabelle7.Cells(9 - 2, t + 3).Value
            If n = 1 Then
```

```
            swap = 0
        End If
    Tabelle7.Cells(9 - 2, t + 3).Value = AandE_Senior + swap
    swap = Tabelle7.Cells(13 - 2, t + 3).Value
        If n = 1 Then
            swap = 0
        End If
    Tabelle7.Cells(13 - 2, t + 3).Value = Ward_1_Senior + swap
    swap = Tabelle7.Cells(17 - 2, t + 3).Value
        If n = 1 Then
            swap = 0
        End If
    Tabelle7.Cells(17 - 2, t + 3).Value = Ward_2_Senior + swap
    swap = Tabelle7.Cells(21 - 2, t + 3).Value
        If n = 1 Then
            swap = 0
        End If
    Tabelle7.Cells(21 - 2, t + 3).Value = Ward_3_Senior + swap
'senior executives
swap = Tabelle7.Cells(9 - 3, t + 3).Value
        If n = 1 Then
            swap = 0
        End If
Tabelle7.Cells(9 - 3, t + 3).Value = AandE_ExecSenior + swap
swap = Tabelle7.Cells(13 - 3, t + 3).Value
        If n = 1 Then
            swap = 0
        End If
Tabelle7.Cells(13 - 3, t + 3).Value = Ward_1_ExecSenior + swap
swap = Tabelle7.Cells(17 - 3, t + 3).Value
        If n = 1 Then
            swap = 0
        End If
Tabelle7.Cells(17 - 3, t + 3).Value = Ward_2_ExecSenior + swap
swap = Tabelle7.Cells(21 - 3, t + 3).Value
        If n = 1 Then
            swap = 0
```

```
                End If
            Tabelle7.Cells(21 - 3, t + 3).Value = Ward_3_ExecSenior + swap
        End If
'..................................
'5.3 update infectious, non-symptoamit staff per unit
    '5.3.1 infectious, non-symptomatic if v_11=0, top down
    If v_11 = 0 Then
        swap = Tabelle6.Cells(54, t + 3).Value
            If n = 1 Then
                swap = 0
            End If
        Tabelle6.Cells(54, t + 3).Value = Staff_pos_nonSymptomatic_AandE + swap
        swap = Tabelle6.Cells(54 + 1, t + 3).Value
            If n = 1 Then
                swap = 0
            End If
        Tabelle6.Cells(54 + 1, t + 3).Value = Staff_pos_nonSymptomatic_ward_1 +
swap
        swap = Tabelle6.Cells(54 + 2, t + 3).Value
            If n = 1 Then
                swap = 0
            End If
        Tabelle6.Cells(54 + 2, t + 3).Value = Staff_pos_nonSymptomatic_ward_2 +
swap
        swap = Tabelle6.Cells(54 + 3, t + 3).Value
            If n = 1 Then
                swap = 0
            End If
        Tabelle6.Cells(54 + 3, t + 3).Value = Staff_pos_nonSymptomatic_ward_3 +
swap
    '5.3.2 infectious, non-symptomatic if v_11=1, exposure focused
    ElseIf v_11 = 1 Then
        swap = Tabelle7.Cells(54, t + 3).Value
            If n = 1 Then
                swap = 0
            End If
        Tabelle7.Cells(54, t + 3).Value = Staff_pos_nonSymptomatic_AandE + swap
```

```
                swap = Tabelle7.Cells(54 + 1, t + 3).Value
                    If n = 1 Then
                        swap = 0
                    End If
                Tabelle7.Cells(54 + 1, t + 3).Value = Staff_pos_nonSymptomatic_ward_1 +
swap
                swap = Tabelle7.Cells(54 + 2, t + 3).Value
                    If n = 1 Then
                        swap = 0
                    End If
                Tabelle7.Cells(54 + 2, t + 3).Value = Staff_pos_nonSymptomatic_ward_2 +
swap
                swap = Tabelle7.Cells(54 + 3, t + 3).Value
                    If n = 1 Then
                        swap = 0
                    End If
                Tabelle7.Cells(54 + 3, t + 3).Value = Staff_pos_nonSymptomatic_ward_3 +
swap
            End If
        '....................................
        '5.4 update infection events staff per unit
            '5.4.1 infection event, top down rollout
            If v_11 = 0 Then
                swap = Tabelle6.Cells(60, t + 3).Value
                    If n = 1 Then
                        swap = 0
                    End If
                Tabelle6.Cells(60, t + 3).Value = Infection_event_AandE + swap
                swap = Tabelle6.Cells(60 + 1, t + 3).Value
                    If n = 1 Then
                        swap = 0
                    End If
                Tabelle6.Cells(60 + 1, t + 3).Value = Infection_event_Ward_1 + swap
                swap = Tabelle6.Cells(60 + 2, t + 3).Value
                    If n = 1 Then
                        swap = 0
                    End If
```

```
            Tabelle6.Cells(60 + 2, t + 3).Value = Infection_event_Ward_2 + swap
            swap = Tabelle6.Cells(60 + 3, t + 3).Value
                If n = 1 Then
                    swap = 0
                End If
            Tabelle6.Cells(60 + 3, t + 3).Value = Infection_event_Ward_3 + swap
        '5.4.2 infection event, exposure focused rollout
        ElseIf v_11 = 1 Then
            swap = Tabelle7.Cells(60, t + 3).Value
                If n = 1 Then
                    swap = 0
                End If
            Tabelle7.Cells(60, t + 3).Value = Infection_event_AandE + swap
            swap = Tabelle7.Cells(60 + 1, t + 3).Value
                If n = 1 Then
                    swap = 0
                End If
            Tabelle7.Cells(60 + 1, t + 3).Value = Infection_event_Ward_1 + swap
            swap = Tabelle7.Cells(60 + 2, t + 3).Value
                If n = 1 Then
                    swap = 0
                End If
            Tabelle7.Cells(60 + 2, t + 3).Value = Infection_event_Ward_2 + swap
            swap = Tabelle7.Cells(60 + 3, t + 3).Value
                If n = 1 Then
                    swap = 0
                End If
            Tabelle7.Cells(60 + 3, t + 3).Value = Infection_event_Ward_3 + swap
        End If


    '...................................
    '5.4 check if unit operational or staff shortage?
    AandE_open = 0
    Ward_1_open = 0
    Ward_2_open = 0
    Ward_3_open = 0
```

```
'A&E partially open
If (AandE_ExecSenior + AandE_Senior > 0) And AandE_Phys > 1 And AandE_Nurse
> 4 Then

    AandE_open = 0.5
End If
    'A&E fully open
    If AandE_Nurse = 10 And AandE_Phys = 4 And AandE_ExecSenior = 1 And
AandE_Senior = 1 Then

        AandE_open = 1
    End If
'ward_1 partially open
If (Ward_1_ExecSenior + Ward_1_Senior > 0) And Ward_1_Phys > 0 And
Ward_1_Nurse > 2 Then

    Ward_1_open = 0.5
End If
    'ward_1 fully open
    If Ward_1_Nurse = 5 And Ward_1_Phys = 2 And Ward_1_ExecSenior = 1 And
Ward_1_Senior = 1 Then

        Ward_1_open = 1
    End If
'ward_2 partially open
If (Ward_2_ExecSenior + Ward_2_Senior > 0) And Ward_2_Phys > 0 And
Ward_2_Nurse > 2 Then

    Ward_2_open = 0.5
End If
    'ward_2 fully open
    If Ward_2_Nurse = 5 And Ward_2_Phys = 2 And Ward_2_ExecSenior = 1 And
Ward_2_Senior = 1 Then

        Ward_2_open = 1
    End If
'ward_3 partially open
If (Ward_3_ExecSenior + Ward_3_Senior > 0) And Ward_3_Phys > 0 And
Ward_3_Nurse > 2 Then

    Ward_3_open = 0.5
End If
    'ward_3 fully open
```

```
                                            If Ward_3_Nurse = 5 And Ward_3_Phys = 2 And Ward_3_ExecSenior = 1 And
Ward_3_Senior = 1 Then
                                                Ward_3_open = 1
                                            End If

                                    '---------------------------------------
                                    'open hospital capacity
                                        'for top down rollout
                                        If v_11 = 0 Then
                                            'current n cycle - write open capacity
                                            Tabelle6.Cells(118 + n, 3 - 1).Value = n
                                            Tabelle6.Cells(118 + n, t + 3).Value = (AandE_open + Ward_1_open +
Ward_2_open + Ward_3_open) / 4

                                            'statistically averaged overview - write open capacity
                                            swap = Tabelle6.Cells(74, t + 3).Value
                                                    If n = 1 Then
                                                        swap = 0
                                                    End If
                                                Tabelle6.Cells(74, t + 3).Value = AandE_open + swap
                                            swap = Tabelle6.Cells(74 + 1, t + 3).Value
                                                    If n = 1 Then
                                                        swap = 0
                                                    End If
                                                Tabelle6.Cells(74 + 1, t + 3).Value = Ward_1_open + swap
                                            swap = Tabelle6.Cells(74 + 2, t + 3).Value
                                                    If n = 1 Then
                                                        swap = 0
                                                    End If
                                                Tabelle6.Cells(74 + 2, t + 3).Value = Ward_2_open + swap
                                            swap = Tabelle6.Cells(74 + 3, t + 3).Value
                                                    If n = 1 Then
                                                        swap = 0
                                                    End If
                                                Tabelle6.Cells(74 + 3, t + 3).Value = Ward_3_open + swap
                                            swap = Tabelle6.Cells(74 + 4, t + 3).Value
                                                    If n = 1 Then
                                                        swap = 0
```

```
                    End If
                Tabelle6.Cells(74 + 4, t + 3).Value = (Ward_1_open + Ward_2_open +
Ward_3_open) / 3 + swap
        'for exposure focused rollout
        ElseIf v_11 = 1 Then
            'current n cycle - write open capacity
            Tabelle7.Cells(118 + n, 3 - 1).Value = n
            Tabelle7.Cells(118 + n, t + 3).Value = (AandE_open + Ward_1_open +
Ward_2_open + Ward_3_open) / 4

            'statistically averaged overview - write open capacity
            swap = Tabelle7.Cells(74, t + 3).Value
                    If n = 1 Then
                        swap = 0
                    End If
                Tabelle7.Cells(74, t + 3).Value = AandE_open + swap
            swap = Tabelle7.Cells(74 + 1, t + 3).Value
                    If n = 1 Then
                        swap = 0
                    End If
                Tabelle7.Cells(74 + 1, t + 3).Value = Ward_1_open + swap
            swap = Tabelle7.Cells(74 + 2, t + 3).Value
                    If n = 1 Then
                        swap = 0
                    End If
                Tabelle7.Cells(74 + 2, t + 3).Value = Ward_2_open + swap
            swap = Tabelle7.Cells(74 + 3, t + 3).Value
                    If n = 1 Then
                        swap = 0
                    End If
                Tabelle7.Cells(74 + 3, t + 3).Value = Ward_3_open + swap
            swap = Tabelle7.Cells(74 + 4, t + 3).Value
                    If n = 1 Then
                        swap = 0
                    End If
                Tabelle7.Cells(74 + 4, t + 3).Value = (Ward_1_open + Ward_2_open +
Ward_3_open) / 3 + swap
        End If
```

```
'#################################################################################################

                                t = t + 1
                                Loop


                    n = n + 1
                    Loop
                        'actual number of loops
                        n = n - 1

                        'write averaged
                            'divide by number of runs
                            'write open capacity
                            t = 1
                            Do While t <= 100
                                    'staff infected top down rollout
                                    If v_11 = 0 Then
                                        swap = Tabelle2.Cells(27, t + 3).Value
                                            Tabelle2.Cells(27, t + 3).Value = swap / n
                                        swap = Tabelle2.Cells(27 + 1, t + 3).Value
                                            Tabelle2.Cells(27 + 1, t + 3).Value = swap / n
                                        swap = Tabelle2.Cells(27 + 2, t + 3).Value
                                            Tabelle2.Cells(27 + 2, t + 3).Value = swap / n
                                        swap = Tabelle2.Cells(27 + 3, t + 3).Value
                                            Tabelle2.Cells(27 + 3, t + 3).Value = swap / n
                                        swap = Tabelle2.Cells(27 + 4, t + 3).Value
                                            Tabelle2.Cells(27 + 4, t + 3).Value = swap / n
                                    'staff infected exposure focused
                                    ElseIf v_11 = 1 Then
                                         swap = Tabelle2.Cells(12, t + 3).Value
                                            Tabelle2.Cells(12, t + 3).Value = swap / n
                                        swap = Tabelle2.Cells(12 + 1, t + 3).Value
                                            Tabelle2.Cells(12 + 1, t + 3).Value = swap / n
                                        swap = Tabelle2.Cells(12 + 2, t + 3).Value
                                            Tabelle2.Cells(12 + 2, t + 3).Value = swap / n
```

```
    swap = Tabelle2.Cells(12 + 3, t + 3).Value
        Tabelle2.Cells(12 + 3, t + 3).Value = swap / n
    swap = Tabelle2.Cells(12 + 4, t + 3).Value
        Tabelle2.Cells(12 + 4, t + 3).Value = swap / n
End If

'staff on units, top down rollout
If v_11 = 0 Then
    swap = Tabelle6.Cells(6, t + 3).Value
        Tabelle6.Cells(6, t + 3).Value = swap / n
    swap = Tabelle6.Cells(6 + 1, t + 3).Value
        Tabelle6.Cells(6 + 1, t + 3).Value = swap / n
    swap = Tabelle6.Cells(6 + 2, t + 3).Value
        Tabelle6.Cells(6 + 2, t + 3).Value = swap / n
    swap = Tabelle6.Cells(6 + 3, t + 3).Value
        Tabelle6.Cells(6 + 3, t + 3).Value = swap / n
    swap = Tabelle6.Cells(6 + 4, t + 3).Value
        Tabelle6.Cells(6 + 4, t + 3).Value = swap / n
    swap = Tabelle6.Cells(6 + 5, t + 3).Value
        Tabelle6.Cells(6 + 5, t + 3).Value = swap / n
    swap = Tabelle6.Cells(6 + 6, t + 3).Value
        Tabelle6.Cells(6 + 6, t + 3).Value = swap / n
    swap = Tabelle6.Cells(6 + 7, t + 3).Value
        Tabelle6.Cells(6 + 7, t + 3).Value = swap / n
    swap = Tabelle6.Cells(6 + 8, t + 3).Value
        Tabelle6.Cells(6 + 8, t + 3).Value = swap / n
    swap = Tabelle6.Cells(6 + 9, t + 3).Value
        Tabelle6.Cells(6 + 9, t + 3).Value = swap / n
    swap = Tabelle6.Cells(6 + 10, t + 3).Value
        Tabelle6.Cells(6 + 10, t + 3).Value = swap / n
    swap = Tabelle6.Cells(6 + 11, t + 3).Value
        Tabelle6.Cells(6 + 11, t + 3).Value = swap / n
    swap = Tabelle6.Cells(6 + 12, t + 3).Value
        Tabelle6.Cells(6 + 12, t + 3).Value = swap / n
    swap = Tabelle6.Cells(6 + 13, t + 3).Value
        Tabelle6.Cells(6 + 13, t + 3).Value = swap / n
    swap = Tabelle6.Cells(6 + 14, t + 3).Value
```

```
        Tabelle6.Cells(6 + 14, t + 3).Value = swap / n
    swap = Tabelle6.Cells(6 + 15, t + 3).Value
        Tabelle6.Cells(6 + 15, t + 3).Value = swap / n
'staff on units, exposure focused
ElseIf v_11 = 1 Then
    swap = Tabelle7.Cells(6, t + 3).Value
        Tabelle7.Cells(6, t + 3).Value = swap / n
    swap = Tabelle7.Cells(6 + 1, t + 3).Value
        Tabelle7.Cells(6 + 1, t + 3).Value = swap / n
    swap = Tabelle7.Cells(6 + 2, t + 3).Value
        Tabelle7.Cells(6 + 2, t + 3).Value = swap / n
    swap = Tabelle7.Cells(6 + 3, t + 3).Value
        Tabelle7.Cells(6 + 3, t + 3).Value = swap / n
    swap = Tabelle7.Cells(6 + 4, t + 3).Value
        Tabelle7.Cells(6 + 4, t + 3).Value = swap / n
    swap = Tabelle7.Cells(6 + 5, t + 3).Value
        Tabelle7.Cells(6 + 5, t + 3).Value = swap / n
    swap = Tabelle7.Cells(6 + 6, t + 3).Value
        Tabelle7.Cells(6 + 6, t + 3).Value = swap / n
    swap = Tabelle7.Cells(6 + 7, t + 3).Value
        Tabelle7.Cells(6 + 7, t + 3).Value = swap / n
    swap = Tabelle7.Cells(6 + 8, t + 3).Value
        Tabelle7.Cells(6 + 8, t + 3).Value = swap / n
    swap = Tabelle7.Cells(6 + 9, t + 3).Value
        Tabelle7.Cells(6 + 9, t + 3).Value = swap / n
    swap = Tabelle7.Cells(6 + 10, t + 3).Value
        Tabelle7.Cells(6 + 10, t + 3).Value = swap / n
    swap = Tabelle7.Cells(6 + 11, t + 3).Value
        Tabelle7.Cells(6 + 11, t + 3).Value = swap / n
    swap = Tabelle7.Cells(6 + 12, t + 3).Value
        Tabelle7.Cells(6 + 12, t + 3).Value = swap / n
    swap = Tabelle7.Cells(6 + 13, t + 3).Value
        Tabelle7.Cells(6 + 13, t + 3).Value = swap / n
    swap = Tabelle7.Cells(6 + 14, t + 3).Value
        Tabelle7.Cells(6 + 14, t + 3).Value = swap / n
    swap = Tabelle7.Cells(6 + 15, t + 3).Value
        Tabelle7.Cells(6 + 15, t + 3).Value = swap / n
```

```
End If

'staff infectious, non-symptomatic on unit, infection events, top down rollout
If v_11 = 0 Then
    swap = Tabelle6.Cells(54, t + 3).Value
        Tabelle6.Cells(54, t + 3).Value = swap / n
    swap = Tabelle6.Cells(54 + 1, t + 3).Value
        Tabelle6.Cells(54 + 1, t + 3).Value = swap / n
    swap = Tabelle6.Cells(54 + 2, t + 3).Value
        Tabelle6.Cells(54 + 2, t + 3).Value = swap / n
    swap = Tabelle6.Cells(54 + 3, t + 3).Value
        Tabelle6.Cells(54 + 3, t + 3).Value = swap / n
    swap = Tabelle6.Cells(60, t + 3).Value
        Tabelle6.Cells(60, t + 3).Value = swap / n
    swap = Tabelle6.Cells(60 + 1, t + 3).Value
        Tabelle6.Cells(60 + 1, t + 3).Value = swap / n
    swap = Tabelle6.Cells(60 + 2, t + 3).Value
        Tabelle6.Cells(60 + 2, t + 3).Value = swap / n
    swap = Tabelle6.Cells(60 + 3, t + 3).Value
        Tabelle6.Cells(60 + 3, t + 3).Value = swap / n
'staff infectious, non-symptomatic on unit, infection events, exposure focused
ElseIf v_11 = 1 Then
    swap = Tabelle7.Cells(54, t + 3).Value
        Tabelle7.Cells(54, t + 3).Value = swap / n
    swap = Tabelle7.Cells(54 + 1, t + 3).Value
        Tabelle7.Cells(54 + 1, t + 3).Value = swap / n
    swap = Tabelle7.Cells(54 + 2, t + 3).Value
        Tabelle7.Cells(54 + 2, t + 3).Value = swap / n
    swap = Tabelle7.Cells(54 + 3, t + 3).Value
        Tabelle7.Cells(54 + 3, t + 3).Value = swap / n
    swap = Tabelle7.Cells(60, t + 3).Value
        Tabelle7.Cells(60, t + 3).Value = swap / n
    swap = Tabelle7.Cells(60 + 1, t + 3).Value
        Tabelle7.Cells(60 + 1, t + 3).Value = swap / n
    swap = Tabelle7.Cells(60 + 2, t + 3).Value
        Tabelle7.Cells(60 + 2, t + 3).Value = swap / n
    swap = Tabelle7.Cells(60 + 3, t + 3).Value
```

```
                    Tabelle7.Cells(60 + 3, t + 3).Value = swap / n
            End If


            'loop unit operational % and divide by number of runs
                'if top down rollout
                If v_11 = 0 Then
                    swap = Tabelle6.Cells(74, t + 3).Value
                        Tabelle6.Cells(74, t + 3).Value = swap / n
                    swap = Tabelle6.Cells(74 + 1, t + 3).Value
                        Tabelle6.Cells(74 + 1, t + 3).Value = swap / n
                    swap = Tabelle6.Cells(74 + 2, t + 3).Value
                        Tabelle6.Cells(74 + 2, t + 3).Value = swap / n
                    swap = Tabelle6.Cells(74 + 3, t + 3).Value
                        Tabelle6.Cells(74 + 3, t + 3).Value = swap / n
                    swap = Tabelle6.Cells(74 + 4, t + 3).Value
                        Tabelle6.Cells(74 + 4, t + 3).Value = swap / n
                ElseIf v_11 = 1 Then 'exposure focused rollout
                    swap = Tabelle7.Cells(74, t + 3).Value
                        Tabelle7.Cells(74, t + 3).Value = swap / n
                    swap = Tabelle7.Cells(74 + 1, t + 3).Value
                        Tabelle7.Cells(74 + 1, t + 3).Value = swap / n
                    swap = Tabelle7.Cells(74 + 2, t + 3).Value
                        Tabelle7.Cells(74 + 2, t + 3).Value = swap / n
                    swap = Tabelle7.Cells(74 + 3, t + 3).Value
                        Tabelle7.Cells(74 + 3, t + 3).Value = swap / n
                    swap = Tabelle7.Cells(74 + 4, t + 3).Value
                        Tabelle7.Cells(74 + 4, t + 3).Value = swap / n
                End If

        t = t + 1
        Loop
    '#######################################################################



'loop parameter variation until stop value reached
```

```
                                'loop both rollout plans
                                v_11 = v_11 + 1
                                Loop While v_11 <= v_11_stop
                                'optimise excel
                                Application.ScreenUpdating = True
                                'save copy
                                Path_xlsm(v_12, v_21, v_22, v_23, v_24, v_25, v_26) = Path_xlsm(v_12, v_21, v_22,
v_23, v_24, v_25, v_26) & "_n_" & n & "-v17.xlsm"
                                ActiveWorkbook.SaveCopyAs Path_xlsm(v_12, v_21, v_22, v_23, v_24, v_25, v_26)
                            v_26 = v_26 + 1
                            Loop While v_26 <= v_26_stop
                        v_25 = v_25 + 1
                        Loop While v_25 <= v_25_stop
                    v_24 = v_24 + 1
                    Loop While v_24 <= v_24_stop
                v_23 = v_23 + 1
                Loop While v_23 <= v_23_stop
            v_22 = v_22 + 1
            Loop While v_22 <= v_22_stop
        v_21 = v_21 + 1
        Loop While v_21 <= v_21_stop
    v_12 = v_12 + 1
    Loop While v_12 <= v_12_stop




End Sub
```
